# Supplementary material for: Prevalence and causes of blindness and vision impairment among people 50 years and older in Nepal: A national Rapid Assessment of Avoidable Blindness survey
Source: PLoS One. 2025 Feb 13;20(2):e0309037. doi: 10.1371/journal.pone.0309037 (PMC11824995; doi:10.1371/journal.pone.0309037)
Supplement: S1 Appendix — (DOCX) [file pone.0309037.s002.docx]

RAAB Nepal Start & end date

Province 01 (Koshi)- 05 June 2019 to September 2019

Province 02 (Madhesh)- 18 December 2019 to 18 December 2020

Province 03 (Bagmati)- 12 June 2019 to December 2019

Province 04 (Gandaki)- 05 June 2029 to October 2019

Province 05 (Lumbini)- 03 October 2018 to December 2018

Province 06 (Karnali)- 03 January 2019 to 02 January 2020

Province 07 (Far western)-19 July 2020 to 19 July 2021
