# Supplementary material for: Prevalence and causes of blindness and vision impairment among people 50 years and older in Nepal: A national Rapid Assessment of Avoidable Blindness survey
Source: PLoS One. 2025 Feb 13;20(2):e0309037. doi: 10.1371/journal.pone.0309037 (PMC11824995; doi:10.1371/journal.pone.0309037)
Supplement: Attachment — Submitted filename: PLOSE One comment_R.docx [file pone.0309037.s003.docx]

PLOS One comment

General comments

I found this manuscript to be well-written overall. I appreciate all the authors who conducted RAAB in Nepal. Some suggestions as follows will help improve further

On page 4, the last line, …the all-age population prevalence was 0.84% in 1981 and 0.35% in 2011. Further in the round bracket, it is written as extrapolated from estimates among those aged 50 and older.

The authors need to check this. Did the author mean the extrapolation was done for the all-age population from the estimates among people aged 50 and above? In this, how was the extrapolation of the population aged less than 50 years done? The author may look up this.

How many provinces are in Nepal? Was the country divided into zones or provinces? It needs some clarification.

Please reframe or improve the writing of the study objectives.

Explain the survey team structure. Mention any pilot study if conducted.

The timeline does not match between the text on page 7 and Table 1. Check it.

On page 8, the data management, the author may mention the specifications of Android devices if recommended by mRAAB developers. Write the full form of CSV at the first appearance.

The author can share how the data confidentiality was ensured if the Amazon Web Services-based- server was used.

In the results, the author used on many fronts …. ‘significantly higher’…. but I don’t see any statistical value or presentation showing p-value in the text. This may be checked. The author can use an alternative word if it is not associated with a statistically indicated significant value.

On page 20, Check the table 3, when the extrapolated magnitude of female and male are added up, it is not match with total extrapolated figure. The author can check it. Other tables seems okay, but re-check it.

The author may present an infographic on the trend of the prevalence of blindness and visual impairment since inception of the national survey in Nepal. This will make readers interested to understand the changes that happened over the years.

Please check the resolution of Figure. Please re-look at the guidelines if any.
