# Supplementary material for: Prevalence and causes of blindness and vision impairment among people 50 years and older in Nepal: A national Rapid Assessment of Avoidable Blindness survey
Source: PLoS One. 2025 Feb 13;20(2):e0309037. doi: 10.1371/journal.pone.0309037 (PMC11824995; doi:10.1371/journal.pone.0309037)
Supplement: Attachment — Submitted filename: Rebuttal Nepal RAAB prevalence 28 Dec2024.docx [file pone.0309037.s005.docx]

1. In the introduction, the authors shared that all-age population prevalence of blindness was extrapolated from the prevalence data of 50 years and older. I think this is not possible at all without employing or knowing the data of people less than 50 years and younger. Hence, would suggest to remove this extrapolation. This extrapolation has been published earlier: Sapkota YD, Limburg H. The epidemiology of blindness in Nepal: 2012. Kathmandu: Nepal Netra Jyoti Sangh. 2012 May 30 https://www.iapb.org/wp-content/uploads/Epidemiology-of-Blindness-Nepal.pdf

2. In abstract statement like 'The prevalence of blindness and any vision impairment were both higher in women than men (1.3% [95% CI 1.1-1.5%] vs 0.9% [95% CI 0.7-1.0%]).' However the data is shown only for blindness. Also statement like 'Cataract was the leading cause of blindness, severe vision impairment and moderate vision impairment while refractive error was the leading cause of mild vision impairment.' is not supported by data in abstract. This has been changed as per reviewer advice. The following sentence has been added to the results section of the abstract: . Cataract was also the leading cause of severe vision impairment (83.9%) and moderate vision impairment (66.8%), while refractive error was the leading cause of mild vision impairment (66.5%)

3. References are still not appropriate. Would appreciate if the authors can go through each reference and ensure they are the right reference and in right place. The references have been edited. Reference 12 : Neena J, Rachel J, Praveen V, Murthy GV, RAAB India Study Group. Rapid assessment of avoidable blindness in India. PloS One 2008;3:e2867. Has been replaced by Vashist P, Senjam SS, Gupta V, Gupta N, Shamanna BR, Wadhwani M, et al. Blindness and visual impairment and their causes in India: Results of a nationally representative survey. PLoS ONE 2022; 17(7): e0271736.

Reviewer #2: All comments addressed. However, I suggest to include the number of people who had fundus examination done. In the examined sample, all those who did not have a dense mature cataract had their fundus examined, there were 17,935 persons of 50 and more years of age who underwent fundus examination.

Reviewer #3: In the results,
So, the words "significant higher" is not meant to be statistically significant since no hypothesis test was done during the analysis. Maybe the author should consider adding one or tow lines on this in the analysis section so no confusion thereby. We have stated in the results section: The prevalence of blindness was significantly higher among females (1.3% [95% CI 1.1 - 1.5%]) than males (0.9% [95% CI 0.7 - 1.0%]); the difference in vision impairment prevalence by sex was not significantly higher at any other threshold of vision impairment (table 3). This has been documented in table 3. Would the reviewer/editorial team advise how this needs to be modified?
