# Supplementary material for: Prevalence and causes of blindness and vision impairment among people 50 years and older in Nepal: A national Rapid Assessment of Avoidable Blindness survey
Source: PLoS One. 2025 Feb 13;20(2):e0309037. doi: 10.1371/journal.pone.0309037 (PMC11824995; doi:10.1371/journal.pone.0309037)
Supplement: Attachment — Submitted filename: Reviewer comments answered 17 Nov 2024.docx [file pone.0309037.s004.docx]

Journal requirements:

When submitting your revision, we need you to address these additional requirements.

1. Please ensure that your manuscript meets PLOS ONE's style requirements, including those for file naming. The PLOS ONE style templates can be found at

https://journals.plos.org/plosone/s/file?id=wjVg/PLOSOne_formatting_sample_main_body.pdf and

https://journals.plos.org/plosone/s/file?id=ba62/PLOSOne_formatting_sample_title_authors_affiliations.pdf

2. Please include a complete copy of PLOS’ questionnaire on inclusivity in global research in your revised manuscript. Our policy for research in this area aims to improve transparency in the reporting of research performed outside of researchers’ own country or community. The policy applies to researchers who have travelled to a different country to conduct research, research with Indigenous populations or their lands, and research on cultural artefacts. The questionnaire can also be requested at the journal’s discretion for any other submissions, even if these conditions are not met.  Please find more information on the policy and a link to download a blank copy of the questionnaire here: https://journals.plos.org/plosone/s/best-practices-in-research-reporting. Please upload a completed version of your questionnaire as Supporting Information when you resubmit your manuscript. The PLOS questionnaire on inclusivity in global research has been added to the submission.

**3. Please note that your Data Availability Statement is currently missing a direct link to access each database. If your manuscript is accepted for publication, you will be asked to provide these details on a very short timeline. We therefore suggest that you provide this information now, though we will not hold up the peer review process if you are unable.**

The datasets are owned by Nepal Netra Jyoti Sangh and are available to download for registered users of the Rapid Assessment of Avoidable Blindness (RAAB) repository via <https://www.raab.world/country-profiles/nepal> (registration is free). Sudurpashchim Province <https://www.doi.org/10.17037/RAABDATASET.00000385>; Gandaki Province <https://www.doi.org/10.17037/RAABDATASET.00000377>; Madhesh Province <https://www.doi.org/10.17037/RAABDATASET.00000379>; Koshi Province <https://www.doi.org/10.17037/RAABDATASET.00000378>; Bagmati Province <https://www.doi.org/10.17037/RAABDATASET.00000360>; Karnali Province <https://www.doi.org/10.17037/RAABDATASET.00000361>; Lumbini Province <https://www.doi.org/10.17037/RAABDATASET.00000349>. The license restricts use of the data for commercial purposes. This restriction has been imposed in accordance with the research ethics approved institutionally by LSHTM and by partner institutions conducting RAABs.

**4. We note that Figures 1 and 2 in your submission contain [map/satellite] images which may be copyrighted. All PLOS content is published under the Creative Commons Attribution License (CC BY 4.0), which means that the manuscript, images, and Supporting Information files will be freely available online, and any third party is permitted to access, download, copy, distribute, and use these materials in any way, even commercially, with proper attribution. For these reasons, we cannot publish previously copyrighted maps or satellite images created using proprietary data, such as Google software (Google Maps, Street View, and Earth). For more information, see our copyright guidelines:** [**http://journals.plos.org/plosone/s/licenses-and-copyright**](http://journals.plos.org/plosone/s/licenses-and-copyright)**.**

The maps in Figures 1 and 2 were created in R by one of the co-authors (IM) using open data from the UN Office for the Coordination of Humanitarian Affairs (OCHA) hosted on Humanitarian Data Exchange (<https://data.humdata.org/dataset/cod-ab-npl>). The data are licenced under a CC BY 3.0 IGO licence (<https://creativecommons.org/licenses/by/3.0/igo/>), therefore, we do not believe that we require written permission to produce the maps for these figures. Please advise.

We require you to either (1) present written permission from the copyright holder to publish these figures specifically under the CC BY 4.0 license, or (2) remove the figures from your submission:

1. You may seek permission from the original copyright holder of Figures 1 and 2 to publish the content specifically under the CC BY 4.0 license.

We recommend that you contact the original copyright holder with the Content Permission Form (http://journals.plos.org/plosone/s/file?id=7c09/content-permission-form.pdf) and the following text:

“I request permission for the open-access journal PLOS ONE to publish XXX under the Creative Commons Attribution License (CCAL) CC BY 4.0 (http://creativecommons.org/licenses/by/4.0/). Please be aware that this license allows unrestricted use and distribution, even commercially, by third parties. Please reply and provide explicit written permission to publish XXX under a CC BY license and complete the attached form.”

Please upload the completed Content Permission Form or other proof of granted permissions as an "Other" file with your submission.

In the figure caption of the copyrighted figure, please include the following text: “Reprinted from [ref] under a CC BY license, with permission from [name of publisher], original copyright [original copyright year].”

2. If you are unable to obtain permission from the original copyright holder to publish these figures under the CC BY 4.0 license or if the copyright holder’s requirements are incompatible with the CC BY 4.0 license, please either i) remove the figure or ii) supply a replacement figure that complies with the CC BY 4.0 license. Please check copyright information on all replacement figures and update the figure caption with source information. If applicable, please specify in the figure caption text when a figure is similar but not identical to the original image and is therefore for illustrative purposes only.

The following resources for replacing copyrighted map figures may be helpful:

USGS National Map Viewer (public domain): http://viewer.nationalmap.gov/viewer/

The Gateway to Astronaut Photography of Earth (public domain): http://eol.jsc.nasa.gov/sseop/clickmap/

Maps at the CIA (public domain): https://www.cia.gov/library/publications/the-world-factbook/index.html and https://www.cia.gov/library/publications/cia-maps-publications/index.html

NASA Earth Observatory (public domain): http://earthobservatory.nasa.gov/

Landsat: http://landsat.visibleearth.nasa.gov/

USGS EROS (Earth Resources Observatory and Science (EROS) Center) (public domain): http://eros.usgs.gov/#

Natural Earth (public domain): http://www.naturalearthdata.com/

5. We note that there is identifying data in the Supporting Information file < RAAB Nepal Start.docx>. Due to the inclusion of these potentially identifying data, we have removed this file from your file inventory. Prior to sharing human research participant data, authors should consult with an ethics committee to ensure data are shared in accordance with participant consent and all applicable local laws.

Individual patient data has not been shared or shown at any point in this manuscript.

Data sharing should never compromise participant privacy. It is therefore not appropriate to publicly share personally identifiable data on human research participants. The following are examples of data that should not be shared:

-Name, initials, physical address

-Ages more specific than whole numbers

-Internet protocol (IP) address

-Specific dates (birth dates, death dates, examination dates, etc.)

-Contact information such as phone number or email address

-Location data

-ID numbers that seem specific (long numbers, include initials, titled “Hospital ID”) rather than random (small numbers in numerical order)

Data that are not directly identifying may also be inappropriate to share, as in combination they can become identifying. For example, data collected from a small group of participants, vulnerable populations, or private groups should not be shared if they involve indirect identifiers (such as sex, ethnicity, location, etc.) that may risk the identification of study participants.

Additional guidance on preparing raw data for publication can be found in our Data Policy (https://journals.plos.org/plosone/s/data-availability#loc-human-research-participant-data-and-other-sensitive-data) and in the following article: http://www.bmj.com/content/340/bmj.c181.long.

Please remove or anonymize all personal information, ensure that the data shared are in accordance with participant consent, and re-upload a fully anonymized data set. Please note that spreadsheet columns with personal information must be removed and not hidden as all hidden columns will appear in the published file.

6. Please include captions for your Supporting Information files at the end of your manuscript, and update any in-text citations to match accordingly. Please see our Supporting Information guidelines for more information: http://journals.plos.org/plosone/s/supporting-information.

**Additional Editor Comments:

The manuscript is an important addition to current understanding on prevalence and causes of blindness and VI in Nepal. However, the authors need to address the below comments as well as the comments from reviewers.**

Thank you for your positive feedback and your time in reviewing this manuscript. **1. The title also need to include causes of blindness and VI.**

Thank you. This has been amended as per reviewer’s suggestions:

“Prevalence and causes of blindness and vision impairment among people 50 years and older in Nepal: a national Rapid Assessment of Avoidable Blindness survey”

**2. Why barriers data was collected for <6/18 and did not include for mild VI.**

This is how it was planned in 2018-19. This manuscript does not present the barriers data.

**3. All the references need to be rechecked once again. For example, The prevalence of blindness was similar to that found in a 2017 RAAB in Bhutan (1.0% [95% CI 0.5-1.4%) but was significantly lower than was found in India in a nationally representative series of RAABs done in 2015-2019 (2.0% [95% CI 1.9–2.1%]).[11, 12] – The reference is not correct**

**The last forty years have seen enormous multi-sectorial efforts to improve eye health in Nepal. The number of ophthalmologists has increased from seven in 1981 to 364 in 2019, while the number of optometrists has increased from zero to 857 and ophthalmic assistants increased from zero to 1,246 in the same period.[8] - The reference is not correct**

Thank you for highlighting these, we have corrected the references.

**4. Multiple typos which need to be corrected.**

A complete spell check has been done again.

**5. Sentences like - Nepal was one of the first countries to endorse the World Health Organization International Agency for the Prevention of Blindness initiative “VISION 2020 The Right to Sight” and, in 2001, developed its first national eye health plan.[3] – Not sure if this is correct.**
This has been edited and rephrased. Nepal endorsed the WHO-IAPB initiative ‘Vision 2020: The Right to Sight’ and in 2001 developed its first national eye health plan.

**Reviewer #1: The studies have been conducted according to standard protocol, and the manuscript is very clearly written.**

Thank you for your positive feedback and your time to review this manuscript.

**Two comments

The survey in the province with least prevalence of blindness and maximum prevalence of blindness have been conducted almost 3 years apart. Do you think that could have influences the results? Looking at the local conditions an opinion could be offered in the discussion section.**

Thank you for raising this interesting point. Covid pandemic happened during the study period and that stretched the timelines as community surveys and door to door campaigning was prohibited for a time. The prevalence in Lumbini (completed 2018) was 1.8% (1.3-2.2) while the prevalence in Sudurpaschim (completed 2021) was 0.7% (0.4-0.9). The prevalence in Sudurpaschim in 2021 was the same as Bagmati (completed 2019) and similar to Koshi (completed 2019) so is not an outlier among the seven provincial results. Given the disruption that Covid caused to service provision in 2020-21, we might anticipate any temporal trend to affect a difference in the opposite direction to what was found here, all other things being equal. Therefore, we think the estimates from the range of years 2018-2021 are reasonably comparable.

We have added the following underlined text to the limitations section:

*“The series of seven surveys took longer to complete than anticipated because data collection was interrupted by the COVID-19 pandemic in 2020. However, we did not find any obvious effect of the pandemic on blindness or VI prevalence in the final province surveyed compared to pre-pandemic results (prevalence was joint lowest among seven here) and believe the provincial findings to be comparable over the duration of the series.”*

**There is a statement that - 'Both Lumbini and Madhesh Provinces have
three major eye hospitals performing high-volume, high-quality cataract surgery; however, more services are needed to meet the need for eye care services of the Nepali population in these provinces as nearly half the hospital workload is catering to patients from neighbouring Indian states.' - Are there any published data/ reports substantiating this? How could you be sure that it is due to capacity limitation and not issues with access in that province.**

The sentence has been re-worded as: 'Both Lumbini and Madhesh Provinces have
three major eye hospitals performing high-volume, high-quality cataract surgery; however, more services are needed to meet the need for eye care services of the Nepali population in these provinces.

**Reviewer #2: RAAB Nepal – Comments**

**Thank you for the opportunity to review the manuscript.** **Please use consistent font and formatting across the manuscript.**

Thank you for your time and your input.

**What was the sample size for each province?**

This information is provided in tables 1 (target sample size per province) and 2 (numbers enrolled and examined per province).

**How many teams were used in each province?**

Each province had three teams, with each team comprising an Ophthalmologist, an Ophthalmic Assistant, and a motivator/data enumerator/cluster informer (Eye Health Worker). Additionally, a hospital-based logistics coordinator was assigned to support the teams. Two teams were deployed at all times, while one backup team was available as needed.

**The duration of data collection varied across the clusters. What are the reasons for this difference?**

Covid pandemic happened during the study period and that stretched the timelines as community surveys and door to door campaigning was prohibited during the pandemic. Also there were differences in sample size, at times there was a limitation in the number of personnel and the travel/logistics challenges were different for the hilly and mountainous regions.

**The duration provided in Table 1 is different from that provided in the text (in red font). Please clarify.** The data in text, red font, refers to the duration of approval given by the ethics committee while the table shows the actual duration of data collection.

**NHRC- Duration of Approval**

Province 01 (Koshi) from 5 June 2019 to 30^th^ September 2019; in Province 02 (Madhesh) from 18 December 2019 to 18 December 2020; Province 03 (Bagmati) from 12 June 2019 to December 2019; in Province 04 (Gandaki) from 05 June 2019 to October 2019; in Province 05 (Lumbini) from 03 October 2018 to December 2018; in Province 06 (Karnali) from 03 January 2019 to 02 January 2020 and in Province 07 (Sudur Paschim) from19 July 2020 to 19 July 2021. We have presented only the data collection dates in the re-submission to avoid confusion.

**Actual Data Collection happened within the duration of Approval**

**Table 1. Sampling and data collection information for each provincial RAAB survey in Nepal 2018-2021.**

| **Province** | **Sampling information** | | | | **Data collection** | |
| --- | --- | --- | --- | --- | --- | --- |
|  | **Estimated prevalence of blindness** | **Population 50 years and older (2011 census)** | **Sample size required** | **Number of clusters (size 35)** | **Data collection period** | **RAAB version used** |
| Koshi | 3.3% | 743,329 | 4360 | 125 | June - September 2019 | 6 |
| Madhesh | 3.5% | 760,911 | 4109 | 118 | Jan- April 2020 | 7 |
| Bagmati | 2.5% | 831,208 | 5800 | 166 | June 2019 - Dec 2019 | 7 |
| Gandaki | 3.0% | 399,059 | 4789 | 137 | Jun-Oct 2019 | 6 |
| Lumbini | 3.0% | 655,927 | 4808 | 138 | Oct-Dec 2018 | 6 |
| Karnali | 2.5% | 190,016 | 4067 | 117 | Jun 2019 - Jan 2020 | 7 |
| Sudur Paschim | 2.9% | 250,982 | 4619 | 132 | Sep 2020 - Feb 2021 | 7 |

**It is unusual to have population-based studies with a response rate close to 100% in a few provinces and also 98% in the overall study. What are the possible reasons for this response rate? Were participants who were not available replaced?**

RAAB survey protocol is not to replace eligible but unavailable participants. RAABs typically have high response rates in spite of this, our response rate is not that unusual. The median response rate for RAABs with this information on the repository is 95% (unpublished, response rates can be reviewed at [www.raab.world](http://www.raab.world)). Sampling the population 50+ with a door-to-door same day enrolment and rapid examination yields high response rates. Further, eligible participants aged 50+ in LMIC, particularly with high proportions of the sample drawn from non-urban populations, tend to work close to their homes, improving their chances of recruitment.

**“Eye with VA worse than 6/12 and no obvious anterior segment pathology were dilated”. How many people were dilated for fundus examination? How was the fundus examination done?**

The number of individuals dilated is not recorded in the RAAB protocol. Fundus examination was done with direct ophthalmoscopy. We have clarified this in the text with the addition of the underlined words.

*“Eyes with presenting VA worse than 6/12 and no obvious anterior segment cause of vision impairment were dilated for fundus examination with direct ophthalmoscopy.”*

**How does the results compare with the recent national survey in the India?**

This comparison was included in the discussion section. The prevalence of blindness was similar to that found in a 2017 RAAB in Bhutan (1.0% [95% CI 0.5-1.4%) but was significantly lower than was found in India in a nationally representative series of RAABs done in 2015-2019 (2.0% [95% CI 1.9–2.1%]).

**The conclusion section is a repetition, please rephrase it.**

The conclusion has been re-phrased as: Cataract was the chief cause of blindness, severe and moderate visual impairment while refractive error was the main cause of mild vision impairment. The prevalence of blindness was more for women than men and varied by province. The Terai, plains region, having the populous Lumbini and Madesh provinces, had a higher prevalence of blindness compared to other five provinces.

**Reviewer #3: PLOS One comment
General comments**

**I found this manuscript to be well-written overall. I appreciate all the authors who conducted RAAB in Nepal. Some suggestions as follows will help improve further**

Many thanks for your positive feedback and time to review this manuscript.

**On page 4, the last line, …the all-age population prevalence was 0.84% in 1981 and 0.35% in 2011. Further in the round bracket, it is written as extrapolated from estimates among those aged 50 and older. The authors need to check this. Did the author mean the extrapolation was done for the all-age population from the estimates among people aged 50 and above? In this, how was the extrapolation of the population aged less than 50 years done? The author may look up this.**

Yes, the all-age estimate of blindness prevalence in 2011 was extrapolated from the estimates in the population aged 50 years and older. The reference provided (#7) explains how this extrapolation was done in some detail. We believe that summarising the methods used here would take several sentences and the detail would prove to be distracting for readers of the introduction, therefore, have not added any additional methodology information to this section. We have clarified the meaning of the text in the brackets with the additional text underlined:

*“The prevalence of blindness and vision impairment in the all-age population was estimated to have decreased from 0.84% in 1981 to 0.35% in 2011 (2011 all-age estimate extrapolated from estimate among people aged 50 years and older).”*

**How many provinces are in Nepal? Was the country divided into zones or provinces? It needs some clarification.**

The country was divided amongst 5 zones earlier (1980s to 2011) and with abolition of monarchy and installation of republic there were seven provinces.

**Please reframe or improve the writing of the study objectives.**

This study aimed to conduct a new nationally representative RAAB, including seven separate surveys of each of seven newly formed provinces in the country. Here, we present vision impairment and blindness estimates for the country and provinces along with the causes of blindness and visual impairment.

**Explain the survey team structure. Mention any pilot study if conducted.**

Each province had three teams, with each team comprising an Ophthalmologist, an Ophthalmic Assistant, and a motivator/data enumerator/cluster informer (Eye Health Worker). Additionally, a hospital-based logistics coordinator was assigned to support the teams. Two teams were deployed at all times, while one backup team was available as needed.

A four-day training session was conducted for the team members by a certified RAAB trainer from the International Centre for Eye Health (ICEH) at the London School of Hygiene and Tropical Medicine (LSHTM). The training included an inter-observer variation (IOV) exercise and a supervised pilot cluster in a non-study area to ensure the teams were familiar with all survey procedures. Each team achieved a minimum Kappa score of 0.6 or higher, demonstrating strong inter-observer agreement on visual acuity, lens status, and the determination of the cause of vision impairment.

**The timeline does not match between the text on page 7 and Table 1. Check it.**

The data in text, in red font on page 7, refers to the duration of approval given by the ethics committee while the table 1 shows the actual duration of data collection.

**On page 8, the data management, the author may mention the specifications of Android devices if recommended by mRAAB developers. Write the full form of CSV at the first appearance.**

Thank you. We have defined CSV as first use here.

**The author can share how the data confidentiality was ensured if the Amazon Web Services-based- server was used.**

We are not entirely sure what the request entails here. The following information is summarised from the RAAB7 website and is also referred to in reference #9 however, we do not believe this level of detail about AWS needs to be included in the manuscript.

*The Amazon Web Services (AWS) Cloud is one of the most secure and well-established cloud computing environments available today. The core AWS infrastructure is built to satisfy the security requirements of healthcare providers, military, global banks, and other high-sensitivity organisations. RAAB7 is hosted on a dedicated AWS Virtual Private Cloud. This provides a secure and isolated section of the AWS Cloud where we have complete control through advanced firewall role based security groups protected by multi-factor-authentication. Security configuration is also validated against the Center for Internet Security AWS Foundations Benchmark, which ensures RAAB7 follows industry-accepted best practices to ensure high-level security configuration. Peek Vision, which developed and hosts the RAAB7 software, is an ISO 27001 certified organisation. ISO 27001 is an internationally recognised security management standard that specifies best practices and robust security controls. The certification requires the implementation of an Information Security Management System that ensures an effective and rigorous security programme which is reviewed and continuously improved over time.*

**In the results, the author used on many fronts …. ‘significantly higher’…. but I don’t see any statistical value or presentation showing p-value in the text. This may be checked. The author can use an alternative word if it is not associated with a statistically indicated significant value.**

This has been done as per reviewer advice. statistically significant difference in subgroup estimates but the study was not powered to detect these so we did not conduct any hypothesis tests.

**On page 20, Check the table 3, when the extrapolated magnitude of female and male are added up, it is not match with total extrapolated figure. The author can check it. Other tables seems okay, but re-check it.**

This is a product of the method used to estimate weighted female, male and total national estimates separately. In the methods section we noted: “The extrapolated magnitude of vision impairment was estimated by applying the age-sex-province weighted prevalence values reported in the paper to the 2021 census counts for female, male, and total populations separately; therefore, male and female estimates do not sum to total estimates.”

**The author may present an infographic on the trend of the prevalence of blindness and visual impairment since inception of the national survey in Nepal. This will make readers interested to understand the changes that happened over the years.**

Sir/Ma’am we have compared our results with results of previous surveys and presented it in Table 6.

**Please check the resolution of Figure. Please re-look at the guidelines if any.**

Thank you. The resolution of the figures had been distorted in the submission process. We have resubmitted the original figures with improved resolution.
